# CBP Bromodomain Inhibition Rescues Mice From Lethal Sepsis Through Blocking HMGB1-Mediated Inflammatory Responses

**DOI:** 10.3389/fimmu.2020.625542

**Published:** 2021-02-02

**Authors:** Xiaowen Bi, Baolin Jiang, Jinyi Zhou, Xirui Fan, Xintong Yan, Juanjuan Liang, Lan Luo, Zhimin Yin

**Affiliations:** ^1^ Jiangsu Province Key Laboratory for Molecular and Medical Biotechnology, College of Life Science, Nanjing Normal University, Nanjing, China; ^2^ State Key Laboratory of Pharmaceutical Biotechnology, School of Life Sciences, Nanjing University, Nanjing, China

**Keywords:** sepsis, CREB binding protein, high mobility group box-1 protein, protein phosphatase 2A, MAPK phosphatase 1

## Abstract

CREB binding protein (CBP), a transcriptional coactivator and acetyltransferase, is involved in the pathogenesis of inflammation-related diseases. High mobility group box-1 protein (HMGB1) is a critical mediator of lethal sepsis, which has prompted investigation for the development of new treatment for inflammation. Here, we report that the potent and selective inhibition of CBP bromodomain by SGC-CBP30 blocks HMGB1-mediated inflammatory responses *in vitro* and *in vivo*. Our data suggest that CBP bromodomain inhibition suppresses LPS-induced expression and release of HMGB1, when the inhibitor was given 8 h post LPS stimulation; moreover, CBP bromodomain inhibition attenuated pro-inflammatory activity of HMGB1. Furthermore, our findings provide evidence that SGC-CBP30 down-regulated rhHMGB1-induced activation of MAPKs and NF-κB signaling by triggering the reactivation of protein phosphatase 2A (PP2A) and the stabilization of MAPK phosphatase 1 (MKP-1). Collectively, these results suggest that CBP bromodomain could serve as a candidate therapeutic target for the treatment of lethal sepsis *via* inhibiting LPS-induced expression and release of HMGB1 and suppressing the pro-inflammatory activity of HMGB1.

## Introduction

CREB binding protein (CBP, also known as CREBBP or KAT3A) was identified as a factor binding to the cAMP response element-binding protein (CREB) (1). CBP is a histone/lysine acetyl-transferase that governs gene expression by modifying chromatin-associated proteins, and has been well known to be implicated in a range of cellular activities, including cell differentiation, proliferation, apoptosis and immune responses (2–4). Furthermore, the bromodomain of CBP is closely related to its biological functions, including the identification of the lysine residues to be acetylated (2). Therapeutic targeting of bromodomains has been recognized as a valuable potential therapy for human malignant inflammatory diseases (5–7). A large number of inhibitors for this target have been reported in the last years, and among them the most potent one is SGC-CBP30, which has been shown to bind with low nanomolar affinity to the bromodomains of CBP (8).

Sepsis is a systemic inflammatory response elicited by microbial infection and the leading cause of mortality in critically ill patients (9). The ultimate cause of death in sepsis is mostly attributed to the multiple organ dysfunction syndrome (MODS) due to uncontrolled inflammatory response to infection or injury, and the commonest sites of infection are the lungs, the liver, kidneys and intestinal tract (10). Many therapeutics attempt treatment of sepsis *via* targeting at tumor necrosis factor alpha (TNF-α) or interleukin (IL)-1. However, anti-TNF-α or anti-IL-1 strategies came in vain to prevent death in animal sepsis models, including LPS injection, bacterial injection, and cecal ligation and puncture (CLP) (11, 12). The reasons for the failure are probably related more to the difficulty in designing clinical trials to inhibit the classic pro-inflammatory cytokines in proper, avoiding damage from the innate immune response. Another non-negligible problem is that patients often come to medical attention relatively late in the disease, and blocking these early cytokines may simply be too late.

High mobility group box-1 protein (HMGB1) has recently been identified as a key “late-phase” mediator and may represent a more effective target for intervention (13, 14). HMGB1 is a non-histone chromosomal protein that can migrate from the nucleus to the cytoplasm, then be actively released by innate immune cells (macrophages or monocytes) under inflammatory or injurious conditions (15). The acetylation modification of HMGB1 plays a central role in its translocation, which is related to the function of CBP as acetyltransferase (16). CBP has the ability to acetylate HMGB1 and its truncated forms lacking the C-terminal domain, and additional acetylation by CBP at Lys81 of truncated HMGB1 resulted in a 3-fold increase of its DNA bending ability (17). Once being released into the extracellular space, HMGB1 further expand the inflammatory responses through stimulating massive production of cytokines. Previous research has shown that HMGB1 can propagate an inflammatory response by binding to receptors (such as RAGE, TLR-2, and TLR-4) to activate MAPKs (mitogen-activated protein kinases), and enhance the expression of proinflammatory cytokines in a NF-κB-dependent mode (18, 19). When monocytes/macrophages were challenged with exogenous bacterial endotoxin, e.g., lipopolysaccharide (LPS), HMGB1 accumulation was first detectable 4 h after stimulation and reached a plateau in expression level around 18 to 24 h (20). Clinically, patients with sepsis have elevated serum levels of HMGB1, and these levels are associated with the severity of organ damage and death (14), suggesting that HMGB1-targeting strategies might be a viable option for clinical trials of sepsis and perhaps other inflammatory diseases.

Numerous studies show that the phosphorylation of MAPKs is balanced by specific MAPK kinases and phosphatases (21). The crosstalk between parallel pathways of the MAPK cascade depends on the expression and activity of protein phosphatases, such as serine/threonine protein phosphatase 2A (PP2A), and MAPK phosphatase 1 (MKP-1), which have been identified to directly dephosphorylate and inactivate JNK, p38 MAPK, and ERK pathway in cells (22, 23). Accumulative data pointing to an important role of PP2A/MKP-1 have been established in a number of disease processes, including inflammatory diseases. PP2A knockout macrophages release more TNF-α upon LPS stimulation (24). MKP-1–deficient mice are highly susceptible to lethal LPS shock (25, 26). In response to LPS, alveolar macrophages from MKP-1–deficient mice show prolonged activation of p38 MAPK and enhanced production of IL-6 and TNF-α (25).

In this study, we show that the inhibitor of CBP prevented sepsis development caused by LPS or CLP, and the combination of ciprofloxacin and SGC-CBP30 had a favorable therapeutic effect on the sepsis model with 80% survival rate. Further *in vitro* experimental data suggested that SGC-CBP30 exerted its therapeutic effects on sepsis by inhibiting the LPS-induced transcriptional output of HMGB1 and its release from THP-1 cells and MPM cells. Furthermore, we demonstrate that the effect of inhibition of CBP bromodomain on preventing rhHMGB1-induced activation of MAPKs and NF-κB pathways *via* reactivating PP2A and maintaining MKP-1 protein stability. These results support CBP bromodomain as an important potential therapeutic target for treatment of lethal sepsis or other inflammatory diseases.

## Materials and Methods

### Antibodies and Reagents

Recombinant human HMGB1 (rhHMGB1, 1690-HMB) and antibody against HMGB1 (MAB16901) were purchased from R&D Systems (Minneapolis, MN, USA). Antibodies against phospho-JNK/SAPK (Thr183/Tyr185) (AP0473) and phospho-p38 MAPK (Thr180/Tyr182) (AP0526) were purchased from ABclonal (Wuhan, Hubei, China). Antibodies against JNK/SAPK (# 9252), p38 MAPK (# 9212), phospho-ERK (Thr202/Tyr204) (# 9101), ERK (# 4695), phosphor-IKKα/β (Ser176/180) (# 2697), IKKα (# 11930), phospho-IκBα (Ser32/36) (# 9246), IκBα (# 9242), phospho-MKP-1 (Ser359) (# 2857), and ubiquitin (# 3936) were purchased from Cell Signaling Technology (Beverly, MA, USA). Antibodies against CBP (ab2832) and PP2A-Cα/β (ab32104) were obtained from Abcam (Cambridge, UK). Antibodies against methyl-PP2A-Cα/β (sc-81603) and MKP-1 (sc-373841) were purchased from Santa Cruz Biotechnology (Dallas, TX, USA). Antibodies against PME-1 (14435-1-AP) and PTPA (10321-1-AP) were purchased from Proteintech (Chicago, IL, USA). DAPI (R37606), Alexa Fluor 555 donkey anti-mouse IgG (A32773) were purchased from Invitrogen (Carlsbad, CA, USA). Antibodies recognizing p-PP2A (Y307) (BS4867), GAPDH (AP0063), Lamin B (AP6001), and β-actin (AP0060) were purchased from Bioworld Technology (Minneapolis, MN, USA). Protease inhibitor MG132 (S2619) and CBP inhibitor SGC-CBP30 (S7256) were purchased from Selleck Chemicals (Houston, TX, USA). PP2A inhibitor okadaic acid (S1786) was purchased from Beyotime Biotechnology (Shanghai, China). MKP-1 inhibitor RO 31-8220 (HY-13866) was purchased from MCE MedchemExpress (New Jersey, USA). Ciprofloxacin (17850), LPS (from *Escherichia coli* O111:B4) (L2630) and X-tremeGENE HP DNA Transfection Reagent (XTG9-RO) were purchased from Sigma-Aldrich (St. Louis, MO, USA).

### Cell Culture

THP-1 cells (human acute monocytic leukemia cell line), obtained from Cell Bank of the Chinese Academic of Sciences (Shanghai, China) were cultured in RPMI 1640 medium (Wisent, 350-000-CL) supplemented with 10% (v/v) fetal bovine serum (Wisent, 086-150) and antibiotics (100 U/ml penicillin and 100 mg/ml streptomycin) (Wisent, 450-201-CL). Primary mouse peritoneal macrophages (MPM) were harvested with serum-free RPMI 1640 medium from eight-week-old male BALB/c mice. The MPM cells were cultured in RPMI 1640 medium supplemented with 15% (v/v) fetal bovine serum and antibiotics (100 U/ml penicillin and 100 mg/ml streptomycin).

### Animal Models of LPS-Induced Endotoxemia and CLP-Induced Sepsis

Eight-week-old male BALB/c mice weighing 20 to 22 g were purchased from Qinglongshan Animal Center (Nanjing, China) [Permit Number: SYXK (Su) 2015-0028]. All the experiments were conducted in accordance with guidelines on the care and use of laboratory animals as outlined in the Provisions and General Recommendation of Chinese Experimental Animals Administration.

The mice were intraperitoneally injected with bacterial endotoxin (LPS, 10 mg/kg) to establish the endotoxemia model. Thirty (30) min or 8 h after LPS challenge, mice were administrated with SGC-CBP30 (7.7 mg/kg, 15.4 mg/kg and 19.3 mg/kg) by intraperitoneal injection. Control animals received similar treatment with sterile saline or dexamethasone (1.3 mg/kg). Mortality was monitored continuously for up to 72 h. Serum HMGB1 and TNF-α levels were detected at 18 h after endotoxemia. In addition, tissues (lungs, colon, liver and kidneys) were excised from the mice 18 h after LPS challenge for histological analysis by staining with hematoxylin and eosin (H&E) and observed under light microscope (Olympus, Japan).

Sepsis was induced in mice by CLP surgery, and severe lethal sepsis was performed with minor modifications to the previously described procedure (27). Mice were anesthetized with sodium pentobarbital (30 mg/kg) before surgery. Hair on mice abdomen was shaved using an electric trimmer and the area was sterilized with alcohol. Under aseptic conditions, the mice were placed onto Styrofoam pads, and a 1 cm incision was made along the midline of the abdomen. The cecum was isolated and ligated at the distal end of the ileocecal valve with a 3-0 silk suture, and punctured twice by piercing the cecum with an 18-gauge needle. The cecum was then squeezed gently to extrude a small amount (droplet) of feces from the perforation sites, and subsequently placed back in the abdomen, and the incision was closed with double sutures. Normal saline (37°C; 5 ml per 100 g body weight) was given subcutaneously to all mice after surgery. Sham surgery that underwent exactly the same procedure except for ligation and perforation of the cecum was conducted on control mice. Then the mice were treated the same as in the LPS-induced endotoxemia model. In the combined therapy with ciprofloxacin (CIP) and SGC-CBP30, CIP (5 mg/kg, i.v.) was administrated to the mice 1 h after CLP surgery and SGC-CBP30 (19.3 mg/kg, i.p.) was administrated to the mice 8 h after CLP surgery.

### Histological Examination

Tissues isolated from mice were fixed in 4% paraformaldehyde, then embedded in paraffin and serially sectioned. The tissue sections were stained with H&E for histological examination. For each mouse, 12 slides were analyzed and the scores were averaged. Measurements were performed in a blind fashion and at the same magnification for all samples.

Lung injuries were evaluated by light microscopic analysis of four parameters including alveolar septal thickness, interstitial edema, infiltration of inflammatory cells, and alveolar congestion/collapse. Each parameter was categorized into four grades: 0 = normal; 1≤ 25%; 2 = 25–50%; 3 = 50–75%; and 4≥75%, and the mean score of the four parameters was used to represent the overall lung injury.

Colon injuries were evaluated by light microscopic analysis of four parameters including bleeding ulcers in the intestinal mucosa, interstitial edema, infiltration of inflammatory cells, and disorganized architecture with intestinal gland. Each parameter was categorized into four grades: 0 = normal; 1≤ 25%; 2 = 25–50%; 3 = 50–75%; and 4≥75%, and the mean score of the four parameters was used to represent the overall colon injury.

Liver injuries were evaluated by light microscopic analysis of four parameters including centrilobular necrosis, hepatocyte edema, infiltration of inflammatory cells, and central venous congestion. Each category was classified into four grades: 0 = normal; 1≤ 25%; 2 = 25–50%; 3 = 50–75%; and 4≥75%, and the mean scores were used to represent the total liver injuries.

Kidney injuries were evaluated by light microscopic analysis of four parameters including the epithelial cell brush-border loss, interstitial edema, infiltration of inflammatory cells, and glomerular shrinkage. Each parameter was categorized into four grades: 0 = normal; 1≤ 25%; 2 = 25–50%; 3 = 50–75%; and 4≥75%, and the mean score of the four parameters was used to represent the overall kidney injury.

### DNA Transfection

Cells were transfected using X-tremeGENE HP transfection reagent with either pcDNA3.1-Flag-PP2Ac, pcDNA3.1-Flag-MKP-1, or the control vector (pcDNA3.1). The total amount of transfected DNA was normalized by addition of empty control plasmids.

### RNA Interference

All shRNA constructs were in the pLKO.1 backbone. Human-specific pLKO.1-CBP shRNA, pLKO.1-PP2A shRNA, and pLKO.1-MKP-1 shRNA were constructed by Public Protein/Plasmid Library (Nanjing, Jiangsu, China). After 72 h of transfection, the transfected cells were treated with stimulators for the indicated time and subjected to immunoblotting analysis.

### Co-Immunoprecipitation and Immunoblotting Analysis

Cells were lysed in a lysis buffer on ice for 30 min, and the lysates were centrifuged at 12,000 g for 15 min. Equal amount of samples were immunoprecipitated with indicated antibodies (normal IgG was used as negative control) at 4 °C overnight, then incubated with protein A/G plus-agarose beads (SC-2003) (Santa Cruz Biotechnology) for an additional 3 h. The immunoprecipitants were separated by SDS-PAGE followed by transferring onto PVDF membranes (Whatman, GE Healthcare, NJ, USA). The membrane was subsequently blocked with 5% nonfat dry milk for 2 h at room temperature (25 °C), followed by incubation with primary antibody overnight at 4 °C, and then incubated with appropriate secondary antibody. Quantification was directly performed on the blot using Tanon Analysis software (Tanon, Shanghai, China).

### Immunofluorescence Microscopy

Cells were fixed with 4% paraformaldehyde for 30 min and permeabilized with 0.2% Triton X-100 for 20 min. After blocking with 5% BSA to eliminate non-specific binding, the cells were incubated with primary antibodies overnight, followed by incubation with FITC-conjugated secondary antibody for 1 h at room temperature (25°C) in the dark. Cells were nuclear-stained with DAPI and examined under Nikon A1 confocal laser microscope system (Tokyo, Japan).

### Measurement of Cytokine Release

The levels of HMGB1, TNF-α, IL-1β, and IL-6 in the serum or culture medium were respectively determined by corresponding ELISA kit, the HMGB1 kit (Mouse:F10620; Human:F01020) were purchased from Westang BioTech (Shanghai, China), kits for TNF-α (Mouse: KGEMC102a; Human: KGEHC103α), IL-1β (Mouse: KGEMC001b; Human: KGEHC002b), and IL-6 (Mouse: KGEMC004; Human: KGEHC007) were purchased from KeyGen BioTech, Jiangsu, China, following the manufacturer’s instructions.

### RNA Preparation and RT-PCR

Total RNA was extracted using RNAiso Plus (9018) (Takara, Shiga, Japan), and cDNA was synthesized using PrimeScript reverse transcription reagents (639506) (Takara, Shiga, Japan). Quantitative real-time PCR was carried out on a LightCycler instrument (Roche, Germany) using SYBR Premix EX Taq reagent (RR600A) (Takara). The housekeeping gene GAPDH was used as an internal control. Primers for specific genes were:

human *HMGB1*, sense: GCGGACAAGGCCCGTTA,
  anti-sense: AGAGGAAGAAGGCCGAAGGA;
human *MKP-1*, sense: CTCCAAGGAGGATATGAAGCG,
  anti-sense: CTCCAGCATCCTTGATGGAGTC;
human *GAPDH*, sense: CGGGAAACTGTGGCGTGAT,
  anti-sense: AGTGGGTGTCGCTGTTGAAGT.

### Statistical Analysis

Experimental data were presented as mean ± SD. All statistical analysis was performed using SPSS 17.0 (SPSS, Chicago, IL, USA). To assess the normality and homogeneity of the results, the Shapiro-Wilk test was performed. To determine statistical differences, Student’s t-test and one-way analysis of variance (ANOVA) followed by Tukey multiple comparisons test were conducted. Kaplan-Meier survival analysis with the log-rank test for between-group comparisons was used to analyze the survival rates of mice. A value of p < 0.05 was considered statistically significant.

## Results

### Treatment With SGC-CBP30 Improved Survival of Sepsis and Decreased the Level of HMGB1 in Serum

**Figure 1:**
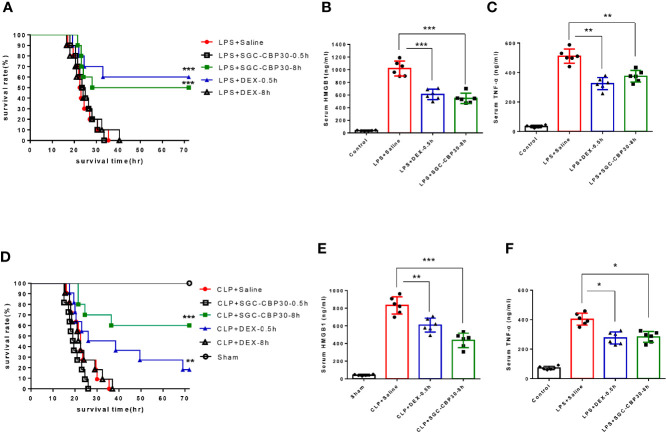
SGC-CBP30 treatment prevented LPS-induced lethal endotoxemia and CLP-induced sepsis. **(A)** Male BALB/c mice received SGC-CBP30 (19.3 mg/kg; intraperitoneal injection) 30 min or 8 h after a lethal dose of LPS (10 mg/kg; intraperitoneal injection). Similar treatments with DEX (1.3 mg/kg; intraperitoneal injection) or saline were taken as control. Mouse survival rate was monitored continuously for 72 h after LPS treatment. Blood was collected after 18 h, and serum HMGB1 **(B)** and TNF-α **(C)** were determined by ELISA. **(D)** Male BALB/c mice were subjected to CLP and SGC-CBP30 (19.3 mg/kg; intraperitoneal injection) was administrated to the mice 30 min or 8 h after surgical procedure. Survival of mice was monitored for 72 h. Serum levels of HMGB1 **(E)** and TNF-α **(F)** 18 h after CLP were measured using ELISA. In **(A, D)**, Kaplan-Meier analysis was used to analyze the survival rate of septic mice. n=10 mice/group. In **(B, C, E, F)**, graphs show mean ± SD. n=6 mice/group. *p < 0.05; **p < 0.01; ***p < 0.001.

As a late inflammatory mediator that contributes to high lethality in sepsis, HMGB1 may be a relevant therapeutic target for intervention. It has been reported that LPS-induced intranuclear CBP protein production can acetylate and thus activate HMGB1, then further stimulate macrophages to produce other inflammatory cytokines such as TNF-α to form an inflammatory cascade (16, 28). To investigate the role of CBP in sepsis pathophysiology, we attempted injecting CBP potent inhibitor SGC-CBP30 after the onset of endotoxemia. Through continuous observation for 72 h after LPS-induced endotoxemia model was established (**Figures 1A**, **S1A**), we found that administration of 19.3 mg/kg SGC-CBP30 at 8 h following LPS challenge (SGC-CBP30-8 h) conferred significant protection against lethality, just slightly behind the positive control DEX-0.5 h therapy group. SGC-CBP30-8 h also simultaneously reduced the serum levels of HMGB1 (**Figure 1B**) and TNF-α (**Figure 1C**). These results show that SGC-CBP30 improved survival by attenuating the production of cytokines in the LPS-induced endotoxemia model.

Although endotoxemia is an animal model available for studying the complex network of cytokines, a more standardized mouse model for severe sepsis is polymicrobial septic condition induced by CLP. At 0.5 or 8 h following the onset of sepsis, mice were intraperitoneally injected with DEX or SGC-CBP30 (**Figures 1D**, **S1B**). It was found that SGC-CBP30-8 h therapy group exhibited higher rescuing rate compared with the positive control DEX-0.5 h therapy group. The effectiveness of delayed administration of SGC-CBP30 for CLP was also confirmed by the serum level of HMGB1 (**Figure 1E**) and TNF-α (**Figure 1F**). These observations indicate that the high mortality rate and uncontrolled proinflammatory responses caused by LPS or CLP correlated with CBP in mice.

### SGC-CBP30 Alleviated Tissue Damage in Sepsis Model

**Figure 2:**
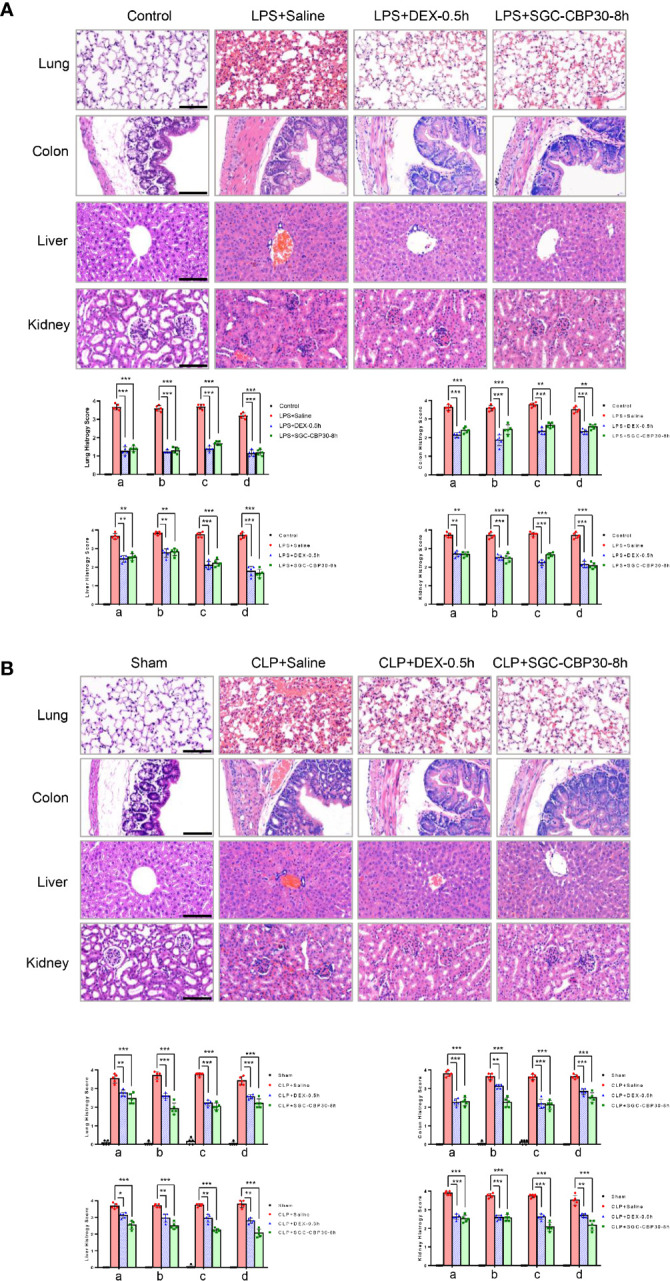
Effect of SGC-CBP30 on tissue damage in sepsis mice. (**A**: LPS-, **B**: CLP-****) Lung tissues, colon tissues, liver tissues, and kidney tissues were collected and subjected to H&E staining 18 h after the models were established, and examined by light microscopy (× 400). Scale bar: 100 μm. Histological scoring of tissue injury were evaluated as described in the *Materials and Methods* section. For lung tissues, the following parameters were evaluated: (a) alveolar septal thickness, (b) interstitial edema, (c) infiltration of inflammatory cells, (d) alveolar congestion/collapse; colon tissues: (a) bleeding ulcers in the intestinal mucosa, (b) interstitial edema, (c) infiltration of inflammatory cells, (d) disorganized architecture with intestinal gland; liver tissues: (a) centrilobular necrosis, (b) hepatocyte edema, (c) infiltration of inflammatory cells, (d) central venous congestion; kidney tissues: (a) epithelial cell brush-border loss, (b) interstitial edema, (c) infiltration of inflammatory cells, (d) glomerular shrinkage. Graphs show mean ± SD. n=5 mice/group. *p < 0.05; **p < 0.01; ***p < 0.001.

Dysregulation of pro-inflammatory cytokines trigger a cytokine storm that may result in tissue damage and organ dysfunction (29). Due to its structural characteristics, lung is the primary failing organ of systemic inflammatory response syndrome (SIRS) (30). Lung injury in septic mice was characterized by alveolar septal thickening, extensive edema, massive infiltration of inflammatory cells, and alveolar congestion/collapse. In lungs from the SGC-CBP30-8 h therapy group, LPS- or CLP-induced histopathological damage and accumulation of inflammatory cells were attenuated (**Figures 2A, B**, first-line). Semi-quantitative assessment of lung histology revealed that administration of SGC-CBP30 at 8 h following LPS or CLP significantly decreased tissue injury in the lung, similar to the positive control DEX-0.5 h.

Systemic sepsis can result in intestinal damage (31). When intestinal injury was assessed using colon specimens stained with H&E, massive histopathological alterations in the colon of septic mice were observed, including intestinal mucosal membrane breakage, colonic edema, massive infiltration of inflammatory cells, and disorganized architecture of the intestine. It was apparent that histological damage and inflammatory cell infiltration were mitigated when treated with SGC-CBP30 at 8 h or DEX at 0.5 h after the induction of endotoxemia and sepsis (**Figures 2A, B**, second line), resulting in a decline in histology score.

Endotoxicosis and sepsis are accompanied by severe dysfunction of liver, which is strongly associated with mortality (32). In mice that had undergone LPS challenge or CLP procedure, centrilobular necrosis, hepatocyte swelling edema, PMN infiltration, and central vein and sinusoidal congestion were observed. In the SGC-CBP30-8 h treatment group, infiltrated inflammatory cells were significantly reduced and liver architecture was significantly improved (**Figures 2A, B**, third-line). As shown in Semi-quantitative statistics, administration of SGC-CBP30 at 8 h or DEX at 0.5 h after septic modeling markedly reduced liver injury score.

Acute kidney injury is a frequent and serious complication of sepsis (33). In terms of histopathological changes, LPS or CLP caused significant deterioration in tubular cells, brush border, and glomerulus. As shown in **Figures 2A, B** (fourth-line), histopathological changes and kidney injury were evident in renal tissues from mice with LPS- or CLP-induced sepsis. It is clear that the delayed SGC-CBP30 administration at 8 h significantly attenuated the histopathological deterioration and inflammatory cell infiltration in renal tissues.

Together, these results demonstrate that selective CBP bromodomain inhibitors such as SGC-CBP30 may be effective not only to suppress inflammatory response in organs, but also to ameliorate organ injury caused by LPS-induced endotoxemia and CLP-induced sepsis.

### SGC-CBP30 Plus Ciprofloxacin Combination Therapy in Sepsis Mice

**Figure 3:**
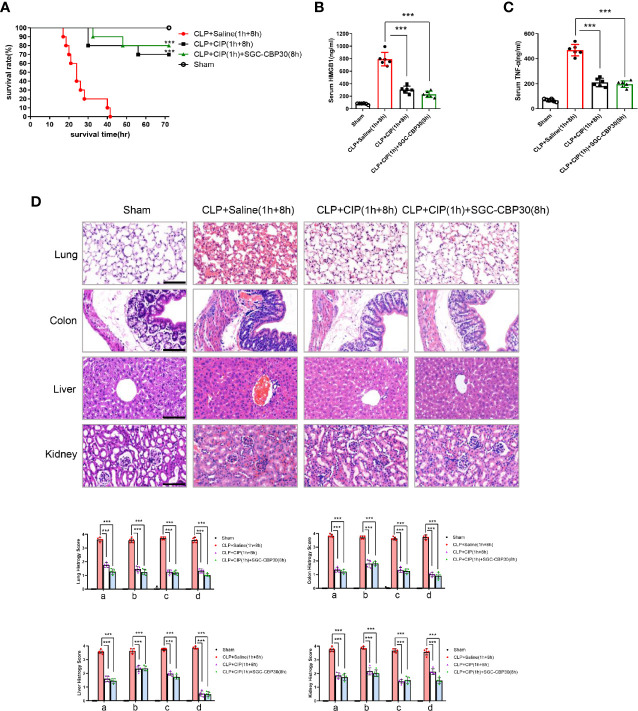
SGC-CBP30 plus ciprofloxacin combination therapy protected mice against lethal sepsis. **(A)** Male BALB/c mice were administered ciprofloxacin (7.5 mg/kg) intravenously at 1 h after CLP, and thereafter administered SGC-CBP30 (19.3 mg/kg) at 8 h after CLP. Survival of mice was monitored for up to 72 h. At 18 h post the onset of sepsis, serum levels of HMGB1 **(B)** and TNF-α **(C)** were determined by ELISA. **(D)** Mice were euthanized 18 h after surgeries and selected organs were collected. Lung, colon, liver, kidney of septic mice was stained with hematoxylin and eosin, and examined by light microscopy (×400). Scale bar: 100 μm. Histological scoring of tissue injury was evaluated as described in the *Materials and Methods* section. For lung tissues, the following parameters were evaluated: (a) alveolar septal thickness, (b) interstitial edema, (c) infiltration of inflammatory cells, (d) alveolar congestion/collapse; colon tissues: (a) bleeding ulcers in the intestinal mucosa, (b) interstitial edema, (c) infiltration of inflammatory cells, (d) disorganized architecture with intestinal gland; liver tissues: (a) centrilobular necrosis, (b) hepatocyte edema, (c) infiltration of inflammatory cells, (d) central venous congestion; kidney tissues: (a) epithelial cell brush-border loss, (b) interstitial edema, (c) infiltration of inflammatory cells, (d) glomerular shrinkage. In (a), Kaplan-Meier analysis was used to analyze the survival rates of septic mice. n=10 mice/group. In **(B–D)**, graphs show mean ± SD. n=5 or 6 mice/group. ***p < 0.001.

Immediate surgical intervention and administration of appropriate antibiotics within 1 h of the identification of severe sepsis are essential to reduce the mortality rate for sepsis (34). However, clinical failures and associated development of antibiotic resistance during therapy have occurred (35). Drug-antibiotic combination has been considered as a way to prevent antibiotic resistance during therapy for sepsis (36). To examine the therapeutic potential of combination treatment with chemical drugs and antibiotics for the therapy of sepsis, ciprofloxacin was administered 1 h after CLP surgery to see whether there were any beneficial effects of SGC-CBP30 in combination with the antibiotic on CLP-induced sepsis. As shown in **Figure 3A**, 70% of the mice were alive in the group treated with ciprofloxacin alone, compared with 80% of mice in the combination therapy group. Cytokines were measured from each treatment group, and it was found that ciprofloxacin treatment reduced the level of HMGB1 in the serum, whereas SGC-CBP30 further increased the inhibitory effect of ciprofloxacin (**Figure 3B**). Meanwhile, treatment with ciprofloxacin alone or in combination with SGC-CBP30 remarkably reduced TNF-α release in CLP mice (**Figure 3C**). In the combination therapy group, the sepsis-caused tissue injury was improved, as reflected by the tissue injury score, which was significantly reduced compared with the sham treatment group (**Figure 3D**), consistent with the lowest level of HMGB1 found in the serum of the combination therapy group.

Together, these observations were the first to demonstrate that treatment with selective CBP inhibitors such as SGC-CBP30 in combination with antibiotics such as ciprofloxacin could dramatically reduce excessive inflammation in sepsis.

### SGC-CBP30 Suppressed LPS-Induced Expression and Extracellular Release of HMGB1

**Figure 4:**
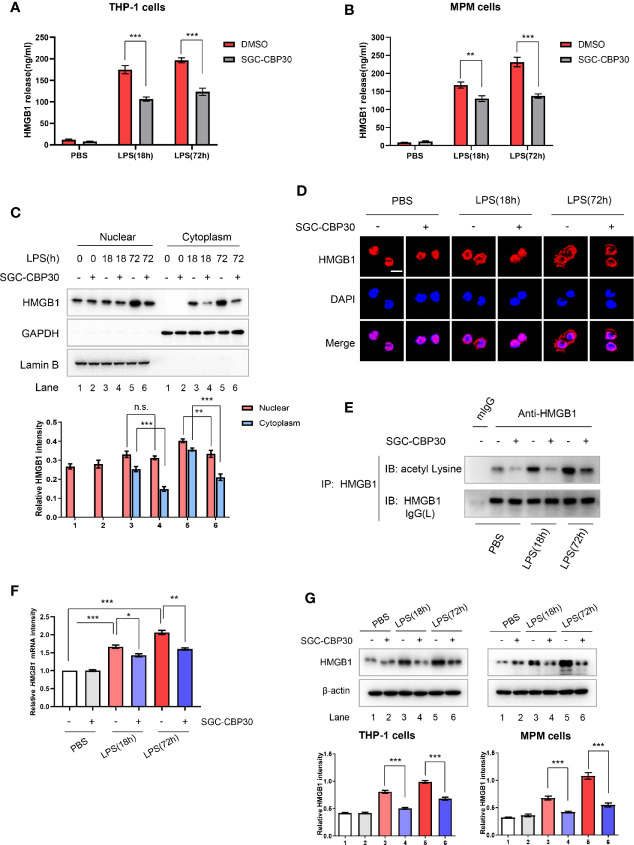
Inhibitory effect of SGC-CBP30 on HMGB1 release and expression in LPS-stimulated cells. THP-1 cells **(A)** and primary MPM cells **(B)** were treated with SGC-CBP30 (4 μM) at 8 h after stimulation with LPS (500 ng/ml). After LPS stimulation for the indicated time, HMGB1 release was measured by ELISA. THP-1 cells were treated with LPS (500 ng/ml) for 8 h and then incubated with SGC-CBP30 (4 μM). After the indicated time of LPS stimulation, nuclear and cytoplasmic fractions were analyzed by Western blot using anti-HMGB1 antibody **(C)**. Cells were incubated with mouse anti-HMGB1 antibody and then incubated with Alexa Flour 555-conjugated anti-mouse (red) secondary antibody. The nuclei were counterstained with DAPI (blue). The location of HMGB1 was observed under a confocal laser microscope. Scale bar: 10 μm **(D)**. The cell lysates were immunoprecipitated with anti-HMGB1 antibody, followed by immunoblotting with anti-acetyl lysine and anti-HMGB1 antibodies **(E)**. Quantification of *HMGB1* transcripts by real-time PCR with GAPDH as the internal control **(F)**. Whole cell lysates were subjected to immunoblotting with anti-HMGB1 and anti-β-actin antibodies **(G)**. Data shown were representative of three independent experiments. Error bars indicate mean ± SD. *p < 0.05; **p < 0.01; ***p < 0.001.

Intrigued by the results of the animal experiments, we performed *in vitro* experiments to investigate how CBP bromodomain affects HMGB1 release. SGC-CBP30, added at 8 h after LPS treatment, decreased LPS-induced HMGB1 release from THP-1 or MPM cells (**Figures 4A, B**). Thus, we next investigated whether SGC-CBP30 affects the cytoplasmic translocation of HMGB1. The results **Figures S2A, B** showed that HMGB1 was predominantly localized in the nucleus under normal conditions, which are in agreement with previous studies (37); and HMGB1 gradually translocated from the nucleus to the cytoplasm in the early stage of LPS stimulation (≤8 h). At the late stage of inflammation (≥18 h), HMGB1 level increased both in the nucleus and in the cytoplasm, probably due to newly synthesized HMGB1 protein. Immunoblotting analysis and confocal microscopy showed that SGC-CBP30 had strong inhibitory effect on LPS-induced high level of cytoplasmic HMGB1 at 18 and 72 h after LPS challenge, while preventing HMGB1 nuclear protein from increasing significantly at 72 h (**Figures 4C, D**). Acetylation of HMGB1 was essential for its translocation from the nucleus to the cytoplasm, as shown in **Figure 4E**, and CBP bromodomain inhibitor SGC-CBP30 significantly diminished the acetylation of HMGB1 in THP-1 cells treated with LPS. In addition, results of RT-PCR (**Figure 4F**) and Western blot (**Figure 4G**) showed that HMGB1 were significantly reduced at both the mRNA and the protein level in cells incubated with SGC-CBP30 at 8 h after LPS stimulation. These results strongly suggested that CBP bromodomain activity is involved in not only the extracellular release, but also the expression of HMGB1.

### CBP Inhibition Attenuated rhHMGB1-Elevated Inflammatory Response

**Figure 5:**
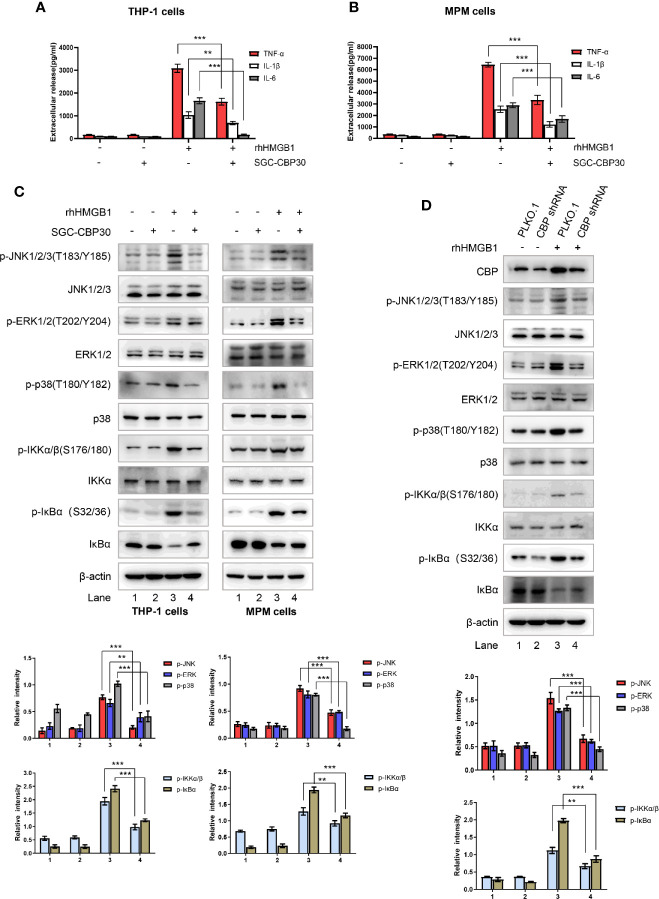
Effects of CBP suppression on HMGB1-mediated inflammatory response. THP-1 cells **(A)** and primary MPM cells **(B)** were pretreated with SGC-CBP30 (4 μM) for 2 h and then stimulated with rhHMGB1 (500 ng/ml) for 12 h; the production of TNF-α, IL-1β, and IL-6 were measured by ELISA. **(C)** THP-1 cells and primary MPM cells were pretreated with or without SGC-CBP30 (4 μM) for 2 h followed by stimulation with rhHMGB1 (100 ng/ml) for 1 h. Cell lysates were prepared and the levels of phospho-MAPKs, phospho-IKKα/β and phospho-IκBα were determined by Western blot analysis. **(D)** THP-1 cells were transfected with CBP shRNA or negative control shRNA, and after 72 h, stimulated with rhHMGB1 (100 ng/ml) for 1 h. The cell lysates were subjected to immunoblotting analysis using indicated antibodies. Data shown were representative of three independent experiments. Error bars indicate mean ± SD. **p < 0.01; ***p < 0.001.

Extracellular HMGB1 could activate a wide range of inflammatory responses including massive production of cytokines such as TNF-α, IL-1β, and IL-6 (14). As shown in **Figures 5A**, **B**, after 12-h stimulation with rhHMGB1, TNF-α, IL-1β, and IL-6 protein secretion were detected. However, the levels of these pro-inflammatory cytokines, which were induced by rhHMGB1, were attenuated after SGC-CBP30 pretreatment. It was reported that HMGB1 binds to RAGE/TLRs and leads to the activation of multiple signaling pathways, including MAPKs and NF-κB (38). Western blotting results demonstrated that the phosphorylation of MAPKs (JNK, ERK, and p38 MAPK), IKKα/β, IκBα and the degradation of IκBα, which were induced by rhHMGB1, were all inhibited by SGC-CBP30 (**Figure 5C**) or by specific shRNA targeting CBP (**Figure 5D**). Together, these results suggest that CBP plays a key role in HMGB1-mediated signaling cascades.

### Inhibition of CBP Bromodomain Prevented rhHMGB1-Induced Inhibition of PP2A Activity and Degradation of MKP-1

**Figure 6:**
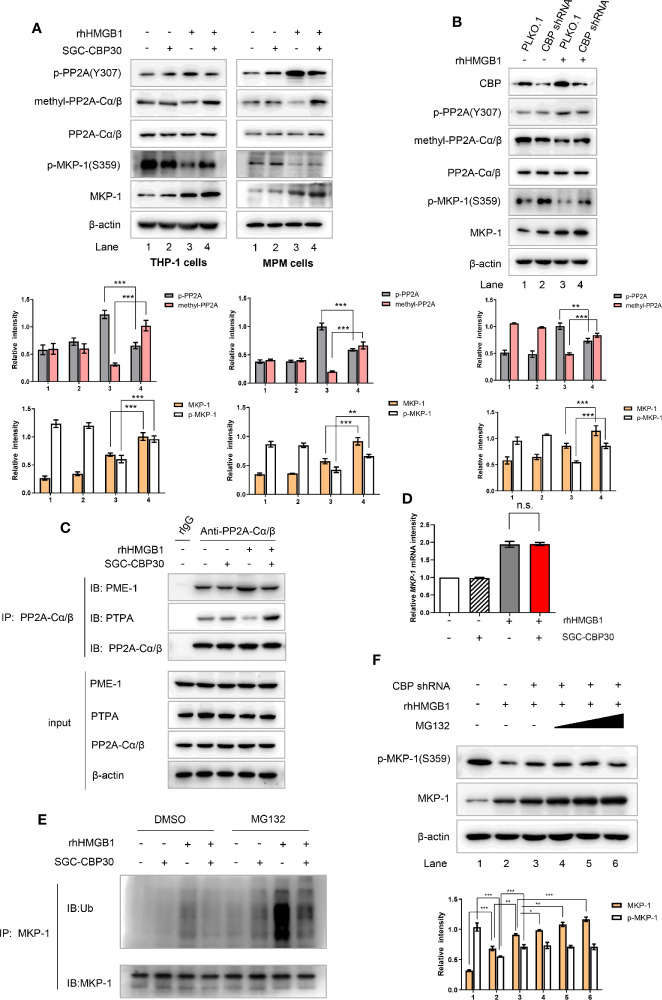
CBP suppression prevented rhHMGB1 from reducing PP2A activity and facilitating MKP-1 degradation. **(A)** THP-1 cells and primary MPM cells were pretreated with SGC-CBP30 (4 μM) for 2 h, followed by rhHMGB1 (100 ng/ml) treatment for 1 h. Whole cell lysates were immunoblotted using anti-CBP, anti-p-PP2A(Y307), anti-methyl-PP2A-Cα/β, anti-PP2A-Cα/β, anti–p-MKP-1(S359) or anti–MKP-1 antibodies, respectively. **(B)** THP-1 cells were transfected with CBP shRNA or negative control shRNA, and 72 h after transfection, cells were stimulated with or without rhHMGB1 (100 ng/ml) for 1 h. Cell lysates were subjected to immunoblotting with indicated antibodies. THP-1 cells were treated the same as in **(A)**, and cell lysates were immunoprecipitated with anti-PP2A-Cα/β then immunoblotted with anti–PME-1 and anti-PTPA antibodies **(C)**. The mRNA expression levels of *MKP-1* were detected by RT-PCR **(D)**. THP-1 cells were pretreated with SGC-CBP30 (4 μM) for 2 h, followed by rhHMGB1 (100 ng/ml) for 1 h in the presence or absence of MG132 (20 μM). Cell lysates were immunoprecipitated using anti–MKP-1 antibody, followed by immunoblotting using antibody against ubiquitin (Ub) **(E)**. Cells were transfected with CBP shRNA, and 72 h after transfection, then treated with or without rhHMGB1 (100 ng/ml) for 1 h in the presence or absence of MG132 (5, 10, or 20 μM). Western blot analysis was performed using anti-p-MKP-1 (S359) or anti-MKP-1 antibodies **(F)**. Data shown were representative of three independent experiments. Error bars indicate mean ± SD. *p < 0.05; **p < 0.01; ***p < 0.001; n.s., no significance.

Protein phosphatases including protein phosphatase 2A (PP2A) and MAPK phosphatase-1 (MKP-1) are well-known negative regulators of MAPKs (39). Therefore, we set out to investigate if the inhibition of CBP bromodomain could down-regulate rhHMGB1 activation of MAPKs by preventing HMGB1 from inhibiting PP2A or MKP-1. To achieve this goal, cells were pre-incubated with SGC-CBP30 to inhibit CBP or with shRNA to knock-down CBP, and then exposed to rhHMGB1. As shown in **Figures 6A, B**, when cells were challenged with rhHMGB1, we detected a rapid decrease in methylated PP2A with an increase in PP2A phosphorylated at Tyr307, both events leading to the inhibition of PP2A activity. Meanwhile, CBP inhibition dramatically reversed rhHMGB1-mediated expression of methylated- and phosphorylated-PP2A, and increased rhHMGB1-induced protein level of MKP-1.

It was shown in mammalian cells that PTPA and PME-1 are parts of a complex mechanism that controls PP2A activity and biogenesis (40). Crystallographic data have suggested that the interaction between PME-1 and PP2A may result in the inactivation of PP2A, while PTPA may reactivate the PME-1–bound inactive form of PP2A in a competitive way (41). In rhHMGB1-stimulated THP-1 cells, the inhibitor SGC-CBP30 specifically inhibited CBP bromodomain to detach PP2A from PME-1; PP2A then bound to the activator PTPA, which in turn enabling PP2A to regain phosphatase activity (**Figure 6C**).

Extracellular stimuli may increase MKP-1 protein level in multiple ways, including transcriptional, post-transcriptional and post-translational. To investigate the mechanism of the high abundance of MKP-1 protein in cells with CBP bromodomain inhibition after rhHMGB1 stimulation, we first assessed the effect of CBP bromodomain on MKP-1 mRNA expression by RT-PCR. As shown in **Figure 6D**, rhHMGB1 induced a significant increase in MKP-1 mRNA level at 1 h, while pre-incubation of SGC-CBP30 had no apparent effect. Phosphorylation modification of MKP-1 contributes to its stability by reducing ubiquitin-mediated proteasomal degradation (42). As expected, inhibition of CBP obviously strengthened rhHMGB1-stimulated p-MKP-1 activation (**Figures 6A, B**), and subsequently resulted in evident reduction in MKP-1 ubiquitination (**Figure 6E**). Moreover, we also observed that proteasome inhibitor MG-132 attenuated MKP-1 degradation (**Figure 6F**).

Collectively, these results implied that CBP bromodomain inhibition prevents rhHMGB1-inhibited PP2A activity, through rescuing methylation of PP2A and attenuating phosphorylation of PP2A; CBP bromodomain inhibition also maintains stabilization of MKP-1 protein.

### SGC-CBP30 Blocked rhHMGB1-Induced MAPKs and NF-κB Activation *via* PP2A-or MKP-1–Dependent Mechanism

**Figure 7:**
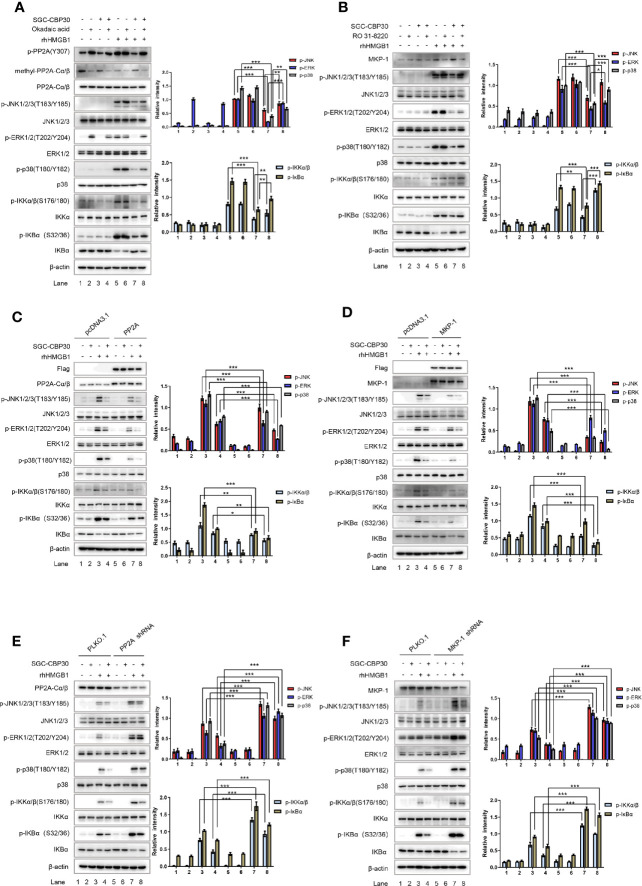
CBP bromodomain inhibitor SGC-CBP30 prevented rhHMGB1-induced inflammatory response by rescuing PP2A/MKP-1. THP-1 cells were pretreated with SGC-CBP30 (4 μM), and then with okadaic acid (100 mM) **(A)** or RO 31-8220 (5 μM) **(B)** for 1 h, followed by treatment with rhHMGB1 (100 ng/ml) for 1 h. Whole cell lysates were subjected to Western blotting using indicated antibodies. Cells were transfected with Flag-PP2A **(C)** or Flag-MKP-1 **(D)** and pretreated with SGC-CBP30 (4 μM) for 2 h, followed by treatment with rhHMGB1 (100 ng/ml) for 1 h. Whole cell lysates were subjected to Western blotting using indicated antibodies. Cells were transfected with PP2A shRNA **(E)** or MKP-1 shRNA **(F)**, followed by pretreatment with SGC-CBP30 (4 μM) for 2 h and then stimulation with rhHMGB1 (100 ng/ml) for 1 h. Whole cell lysates were subjected to Western blotting using indicated antibodies. Data shown were representative of three independent experiments. Error bars indicate mean ± SD. *p < 0.05; **p < 0.01; ***p < 0.001.

To pinpoint the role of PP2A and MKP-1 in CBP bromodomain regulation of rhHMGB1-induced MAPKs and NF-κB activation, we next employed PP2A inhibitor okadaic acid and MKP-1 inhibitor RO 31-8220 in the experiments. As shown in **Figure 7A**, co-treatment with SGC-CBP30 and okadaic acid significantly reversed the inhibitory effect of SGC-CBP30 alone on rhHMGB1-stimulated events, among which ERK pathway was the most obvious one. RO 31-8220 had similar effects in attenuating the inhibitory effect of SGC-CBP30 on MAPKs and NF-κB activation; however, in contrast to p38 MAPK and JNK, the restoration in ERK activity was minimal (**Figure 7B**).

To confirm the roles of PP2A and MKP-1 in SGC-CBP30 inhibition of rhHMGB1-induced activation of MAPKs and NF-κB, THP-1 cells were transfected with Flag-PP2A or Flag-MKP-1, respectively. Western blot showed that overexpression of PP2A or MKP-1 conferred partial resistance to rhHMGB1-triggered activation of MAPKs and NF-κB, and potentiated the inhibitory effect of SGC-CBP30 on the events (**Figures 7C, D**).

To find out why the activity of PP2A and the level of MKP-1 is responsible for the inhibitory effects of SGC-CBP30 on rhHMGB1-induced signaling cascades, we carried out gene silencing experiments. As shown in **Figures 7E, F**, after treatment with rhHMGB1, a higher basal level of MAPKs and NF-κB activation could be detected in cells infected with shRNA targeting PP2A or MKP-1 (compared with PLKO.1-infected cells). As expected, due to failure to activate PP2A in cells expressing shRNA-PP2A, SGC-CBP30 did not significantly inhibit rhHMGB1-induced phospho-ERK1/2 (**Figure 7E**). In addition, infection with shRNA-MKP-1 markedly relieved the decrease of MAPKs and NF-κB activation in SGC-CBP30 treatment group (**Figure 7F**).

Taken together, our data support the concept that inhibition of CBP bromodomain prevents rhHMGB1-induced inflammatory response by rescuing PP2A/MKP-1, thus blocking the activation of the MAPKs and NF-κB cascade.

## Discussion

Many studies have shown that the incidence of sepsis is a complex clinical syndrome with multiple factors involved in the pathological process, but the exact pathogenesis remains incompletely understood. In the present study, we demonstrated that inhibition of CBP bromodomain at 8 h following the onset of sepsis by the selective and potent inhibitor SGC-CBP30 significantly increased the survival rate of mice with severe sepsis, at least in part, through a mechanism that involves expression, active release, and the pro-inflammatory activity of HMGB1. Bromodomain was discovered in the early 1990s in the *brahma* gene from *Drosophila melanogaster*, which primarily initiates the recognition of ϵ−N−acetylation of lysine residues (6, 43). The transcriptional coactivator CBP possesses such a bromodomain, and small-molecule therapies targeting the CBP bromodomain were validated as a strategy to inhibit growth of tumor cell lines and promote cellular senescence (5, 7, 8). More recently, based on the results obtained from *in vivo* and *in vitro* models, it has been suggested that CBP bromodomain inhibition can be used in other pathophysiological conditions such as immune response (7).

Sepsis is a systemic inflammatory syndrome that can lead to lethal organ damage, especially the lungs, colon, liver, and kidneys (10). The biggest obstacle to the discovery of therapeutics for sepsis is that its pathogenesis remains obscure. However, it is known that its regulation is achieved in part by the levels of HMGB1 accumulation. Previous studies have shown that administration of HMGB1 to experimental mice caused lethal organ damage, while passive immunization with neutralizing anti-HMGB1 antibodies before or after endotoxin exposure reversed the lethality of established sepsis, and decreased organ injury in mice subjected to severe sepsis (44). In this study, we have shown that SGC-CBP30 attenuated circulating HMGB1 levels even when the treatment was started at 8 h after sepsis modeling, and that HMGB1 induced the release of TNF-α, suggesting that CBP bromodomain might function as a regulator in controlling HMGB1. These findings were consistent with previous studies on the efficacy of CBP in acetylating intranuclear HMGB1, which is involved in the active release of HMGB1 (16). Although the reason for no therapeutic effect of SGC-CBP30-0.5 h therapy group remains to be elucidated, it can be predicted that administrated with SGC-CBP30 at 0.5 h post sepsis modeling, it is difficult to maintain effective blood concentrations to combat the onset of HMGB1-mediated inflammatory storms at the “late-phase” of lethal sepsis. In addition, timely administration of appropriate antimicrobial therapy is required to halt the progression of systemic inflammatory syndrome (34). Interestingly, ciprofloxacin and SGC-CBP30 combination therapy has a satisfying therapeutic effect against sepsis, as shown by the survival and sepsis-related organ dysfunction data. Although survival was not different statistically between monotherapy and combination therapy, the additional survival and multiple organ dysfunction benefit with combination therapy may be clinically meaningful in severe sepsis, which lacks appropriate early identification criteria.

Stimulation of monocytes or macrophages by lipopolysaccharides or inflammatory cytokines leads to HMGB1 translocation from the nucleus into the cytoplasm (45). Meanwhile, an obvious increase in gene expression of HMGB1 mRNA was detected 24 h after stimulation (16). Our *in vitro* studies on THP-1 cells and primary MPM cells indicated that SGC-CBP30 decreased not only the LPS-induced HMGB1 nucleocytoplasmic transport, but also the synthesis of HMGB1. CBP is endowed with acetyltransferase activity, which transfers an acetyl group to the ϵ-amino group of a lysine residue in histone tails and other nuclear proteins, thereby promoting transcription and enhancing gene activity (2, 3). And the acetyl-lysine specific CBP bromodomain is required for the maintenance of CBP enzymatic activity (2). In the present study, we provided a valuable clue that CBP bromodomain inhibitors such as SGC-CBP30 might be used as therapeutic agents for sepsis, which is regulated by HMGB1.

Once released from stimulated monocytes or macrophages, extracellular HMGB1 employs several cell surface receptors (such as TLR2, TLR4, or RAGE) to activate innate immune cells, causing “second attack” to the body (18, 28). In the present study, we demonstrated that CBP inhibition suppressed rhHMGB1 triggered MAPKs and NF-κB signaling, and in turn, attenuated HMGB1 activity in the production of pro-inflammatory cytokines. Likewise, in the SGC-CBP30 treated sepsis model, the second wave of serum TNF-α level was dramatically reduced. Accumulating studies have shown that PP2A and MKP-1 are linked to MAPKs inactivation (22, 23, 39). Here, we found that rhHMGB1 treatment induced inactivation of PP2A and ubiquitin-dependent degradation of MKP-1, resulting in activation of MAPKs and NF-κB, which may be mediated by the CBP bromodomain. It appeared that the patterns of CBP bromodomain in regulating PP2A and MKP-1 were different. CBP inhibition did not affect the protein level of PP2A, but suppressed rhHMGB1-induced inactivation of PP2A *via* PTPA competitive binding of PP2A from inactive PP2A/PME-1 complex. In contrast, an increased level of MKP-1 was detected by attenuation of MKP-1 protein degradation after treatment with SGC-CBP30.

To test the hypothesis that CBP inhibition prevents the pro-inflammatory activity of HMGB1 *via* PP2A/MKP-1–dependent mechanism in monocytes and macrophages, pharmacological/genetic inhibition or rescue studies for PP2A and MKP-1 were carried out, respectively. Our study showed that okadaic acid (PP2A inhibitor), RO 31-8220 (MKP-1 inhibitor), and PP2A or MKP-1 shRNA resulted in potent resistance to SGC-CBP30 inhibition of rhHMGB1-stimulated phosphorylation of MAPKs and NF-κB. In contrast, overexpression of wild-type PP2A or MKP-1 dramatically inhibited rhHMGB1-induced phosphorylation of MAPKs and NF-κB in the presence or absence of SGC-CBP30. Thus, we have identified that therapeutic targeting of CBP bromodomain is likely to act by mechanisms that counteract HMGB1-mediated inactivation of PP2A and degradation of MKP-1, thereby preventing rhHMGB1-elevated MAPKs and NF-κB signaling. Further *in vivo* experiments conducted in BALB/c mice administered with toxic doses of purified mouse recombinant HMGB1 would help to more thoroughly understand the role of CBP in regulating the pro-inflammatory activity of HMGB1.

**Figure 8:**
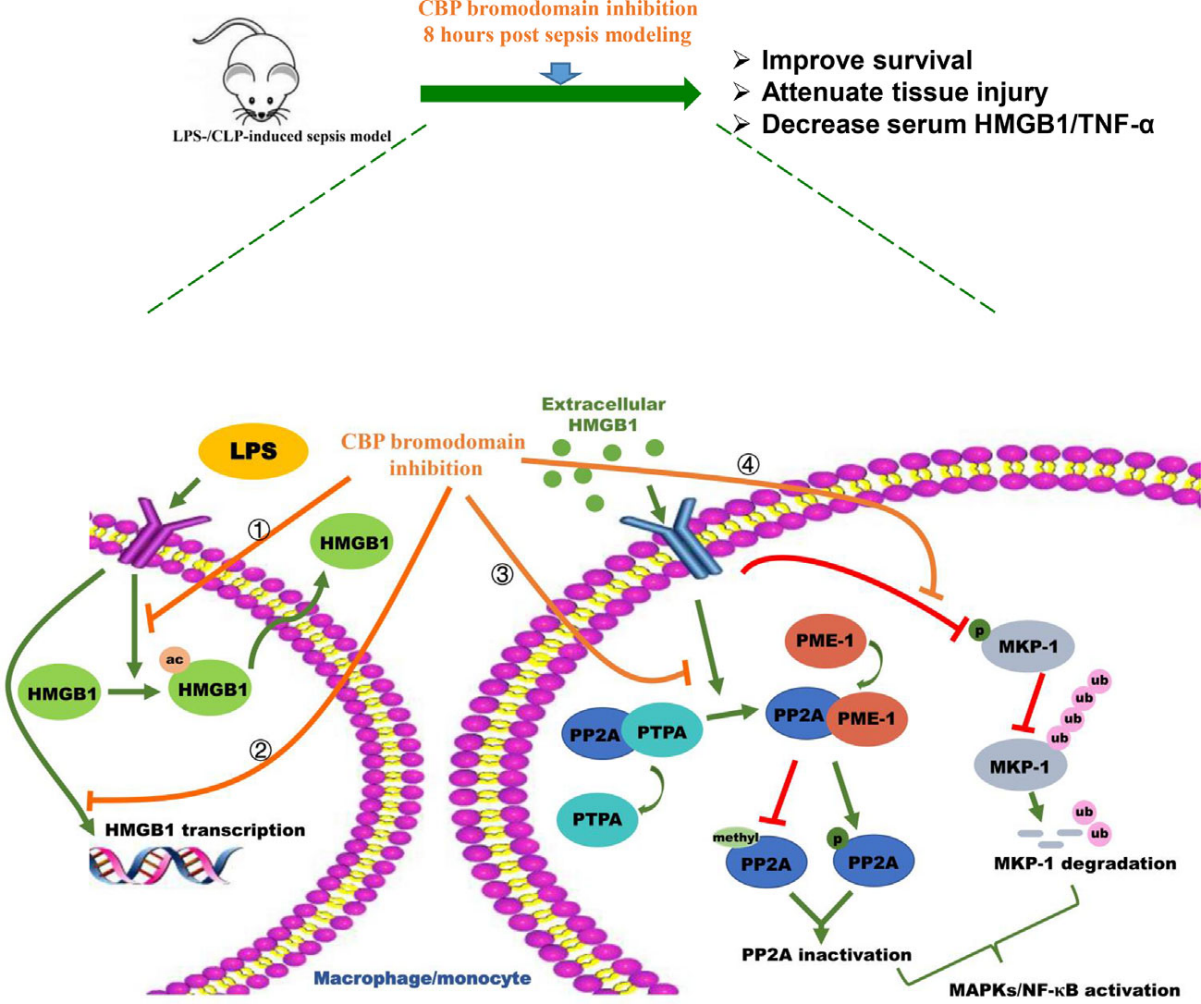
Schematic illustration of CBP bromodomain inhibitor prevents sepsis-related HMGB1 secretion and blocks HMGB1 pro-inflammatory activity. The potent and selective CBP inhibitor, SGC-CBP30, significantly rescues mice from LPS-/CLP-induced sepsis model. In addition, SGC-CBP30 suppresses LPS-induced HMGB1 expression and cytoplasmic translocation. Furthermore, by triggering the reactivation of PP2A and the stabilization of MKP-1, CBP bromodomain inhibition prevents rhHMGB1-stimulated activation of MAPKs and NF-κB pathways and production of proinflammatory cytokines.

In conclusion, the *in vivo* work on LPS- and CLP-induced sepsis mice demonstrates that CBP bromodomain maybe a therapeutic target for sepsis, and the most potent CBP inhibitor, SGC-CBP30, significantly rescued mice from the sepsis model. In addition, our *in vitro* studies indicate that SGC-CBP30 abrogated LPS-induced HMGB1 expression and cytoplasmic translocation. Furthermore, CBP is a critical protein in rhHMGB1-stimulated pro-inflammation response by inactivation of PP2A and destabilization of MKP-1 in THP-1 and MPM cells. By rescuing PP2A and MKP-1, CBP bromodomain inhibition prevents rhHMGB1-stimulated activation of MAPKs and NF-κB pathways and production of proinflammatory cytokines (**Figure 8**). Our findings indicate that pharmacological targeting of CBP bromodomain represents a possible treatment to control lethal sepsis or other inflammatory diseases.

## Data Availability Statement

The raw data supporting the conclusions of this article will be made available by the authors, without undue reservation.

## Ethics Statement

The animal study was reviewed and approved by Experimental Animal Center of Nanjing Normal University.

## Author Contributions

Conceived and designed the experiments: ZY, LL, and XB. Performed the experiments: XB, BJ, JZ, XF, XY, and JL. Analyzed the data: XB, ZY, and LL. Wrote the paper: XB and ZY. All authors contributed to the article and approved the submitted version.

## Funding

This work was financially supported by grants from the Natural Science Foundation of China (32001023, 81671565, and 81771703), the China Postdoctoral Science Foundation (2020T130058ZX), the Priority Academic Program Development of Jiangsu Higher Education Institution (PADD).

## Conflict of Interest

The authors declare that the research was conducted in the absence of any commercial or financial relationships that could be construed as a potential conflict of interest.

## Supplementary Material

Supplementary Figure 1 The mortality rate of sepsis mice with SGC-CBP30 treatment. Male BALB/c mice were treatment with 7.7 mg/kg, 15.4 mg/kg or 19.3 mg/kg of SGC-CBP30, 8 h after LPS (10 mg/kg) challenge **(A)**; 7.7 mg/kg, 15.4 mg/kg or 19.3 mg/kg of SGC-CBP30 was administrated to the mice 8 h after CLP surgery **(B)**, and the survival rate of mice was observed continuously. Kaplan-Meier analysis was used to analyze the survival rate of septic mice. n=10 mice/group. **p < 0.01; ***p < 0.001.

Supplementary Figure 2 LPS-induced HMGB1 expression and translocation. THP-1 cells were treated with LPS (500 ng/ml) for 18 h or 72 h, and after LPS stimulation, nuclear and cytoplasmic fractions were subjected to Western blot with antibody against HMGB1 **(A)**. The mRNA expression level of *HMGB1* was detected by real-time PCR analysis **(B)**. Data shown were representative of three independent experiments. Error bars indicate mean ± SD. ***p < 0.001; n.s., no significance.
