# Supplementary figures and images for: CBP Bromodomain Inhibition Rescues Mice From Lethal Sepsis Through Blocking HMGB1-Mediated Inflammatory Responses
Source: Front Immunol. 2021 Feb 2;11:625542. doi: 10.3389/fimmu.2020.625542 (PMC7884462; doi:10.3389/fimmu.2020.625542)

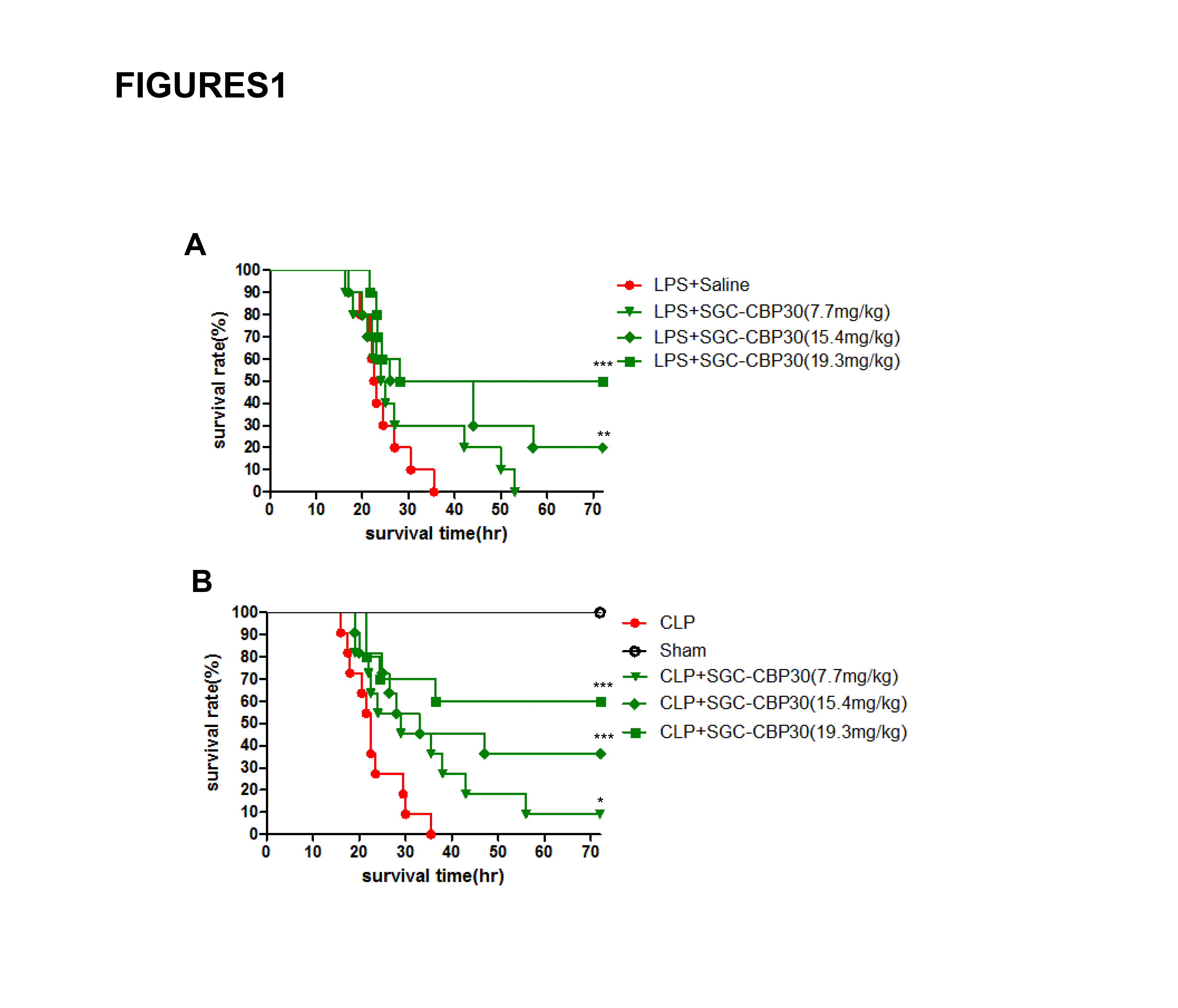

Supplement: Supplementary Figure 1 — The mortality rate of sepsis mice with SGC-CBP30 treatment. Male BALB/c mice were treatment with 7.7 mg/kg, 15.4 mg/kg or 19.3 mg/kg of SGC-CBP30, 8 h after LPS (10 mg/kg) challenge (A); 7.7 mg/kg, 15.4 mg/kg or 19.3 mg/kg of SGC-CBP30 was administrated to the mice 8 h after CLP surgery (B), and the survival rate of mice was observed continuously. Kaplan-Meier analysis was used to analyze the survival rate of septic mice. n=10 mice/group. **p < 0.01; ***p < 0.001. [file Image_1.tif]

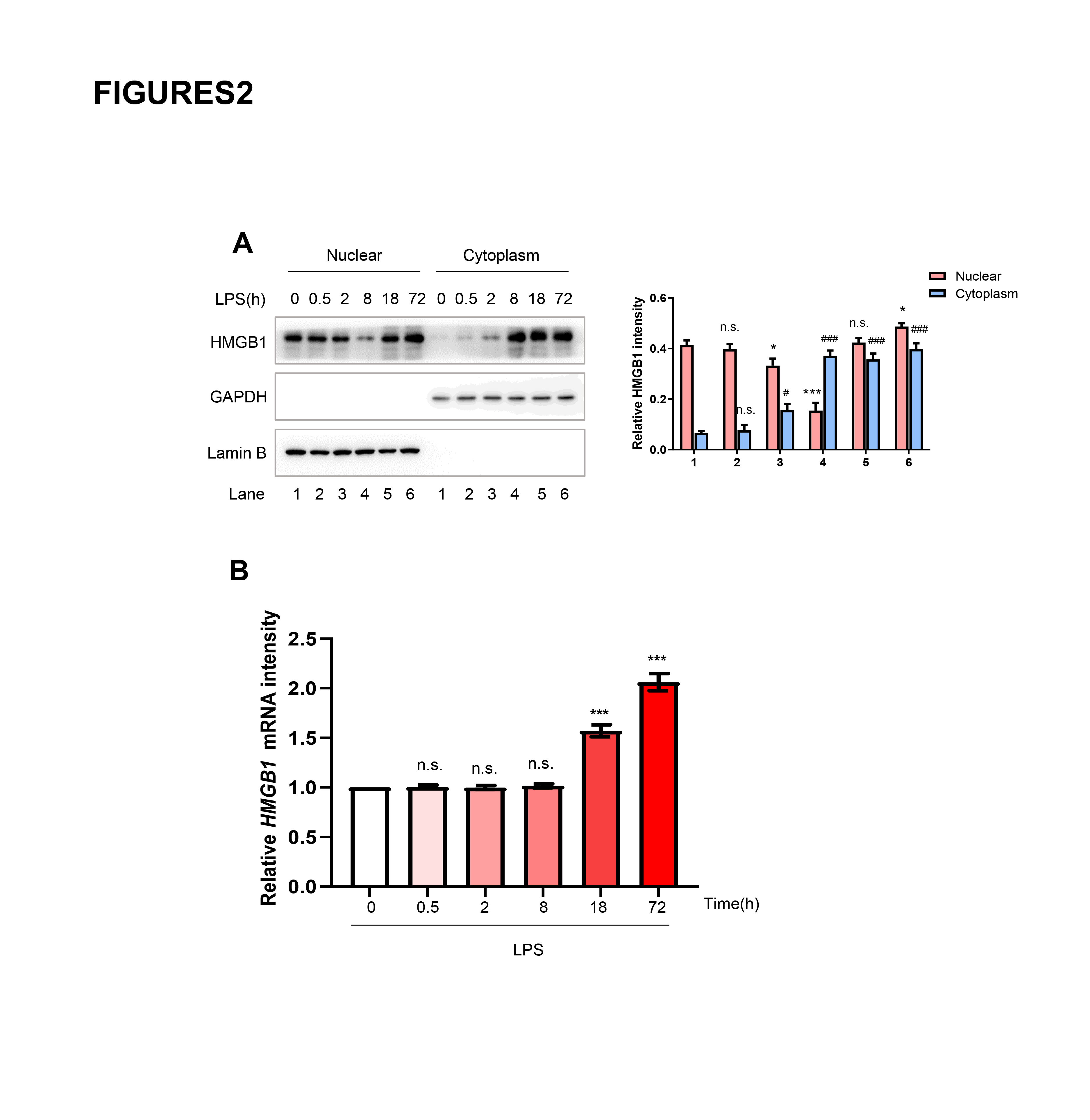

Supplement: Supplementary Figure 2 — LPS-induced HMGB1 expression and translocation. THP-1 cells were treated with LPS (500 ng/ml) for 18 h or 72 h, and after LPS stimulation, nuclear and cytoplasmic fractions were subjected to Western blot with antibody against HMGB1 (A). The mRNA expression level of HMGB1 was detected by real-time PCR analysis (B). Data shown were representative of three independent experiments. Error bars indicate mean ± SD. ***p < 0.001; n.s., no significance. [file Image_2.tif]
